# Garlic and Chitosan Improve the Microbial Quality of Hummus and Reduce Lipid Oxidation

**DOI:** 10.3390/foods13244074

**Published:** 2024-12-17

**Authors:** Tareq M. Osaili, Anas A. Al-Nabulsi, Asma’ O. Taybeh, Amin N. Olaimat, Sadi Taha, Layal Karam, Mutamed Ayyash, Fayeza Hasan, Maher M. Al Dabbas, Gafar Babatunde Bamigbade, Murad Al-Holy, Ioannis N. Savvaidis, Reyad S. Obaid, Richard Holley

**Affiliations:** 1 Department of Clinical Nutrition and Dietetics, College of Health Sciences, The University of Sharjah, Sharjah P.O. Box 27272, United Arab Emirates; robaid@sharjah.ac.ae; 2 Research Institute for Medical and Health Sciences, University of Sharjah, Sharjah P.O. Box 27272, United Arab Emirates; u00040620@sharjah.ac.ae; 3 Department of Nutrition and Food Technology, Faculty of Agriculture, Jordan University of Science and Technology, P.O. Box 3030, Irbid 22110, Jordan; anas_nabulsi@just.edu.jo (A.A.A.-N.); ataybeh@just.edu.jo (A.O.T.); 4 Department of Clinical Nutrition and Dietetics, Faculty of Applied Medical Sciences, The Hashemite University, P.O. Box 150459, Zarqa 13115, Jordan; aminolaimat@hu.edu.jo (A.N.O.); murad@hu.edu.jo (M.A.-H.); 5 Nutrition and Food Processing Department, Al-Huson University College, Al-Balqa Applied University, Irbid 21510, Jordan; saditaha.smstraining@gmail.com; 6 Department of Nutrition Sciences, College of Health Sciences, QU Health, Qatar University, Doha P.O. Box 2713, Qatar; lkaram@qu.edu.qa; 7 Department of Food Science, College of Agriculture & Veterinary Medicine, United Arab Emirates University (UAEU), Al Ain P.O. Box 15551, United Arab Emirates; mutamed.ayyash@uaeu.ac.ae (M.A.); gbamigbade@uaeu.ac.ae (G.B.B.); 8 Department of Nutrition and Dietetics, College of Pharmacy, Alain University, Abu Dhabi P.O. Box 6414, United Arab Emirates; maher.dabbas@aau.ac.ae; 9 Department of Nutrition and Food Technology, Faculty of Agriculture, The University of Jordan, Amman 11942, Jordan; 10 Department of Environmental Health Sciences, College of Health Sciences, University of Sharjah, Sharjah P.O. Box 27272, United Arab Emirates; 11 Department of Chemistry, University of Ioannina, 45110 Ioannina, Greece; 12 Department of Food Science and Human Nutrition, University of Manitoba, Winnipeg, MB R3T 2N2, Canada; rholley@umanitoba.ca

**Keywords:** chickpea dip, spoilage bacteria, natural antimicrobials, shelf life, ready-to-eat foods

## Abstract

This study investigated the antimicrobial and antioxidant effects of garlic and chitosan on hummus. Hummus was prepared by using 0.5% or 1% (*w*/*w*) chitosan, with or without 1% (*w*/*w*) garlic, and samples were stored at 4, 10, or 25 °C for 28, 21, or 7 d, respectively. The behavior of lactic acid bacteria (LAB), *Pseudomonas* spp., aerobic bacteria, and yeasts and molds was then investigated. Color, pH, TBARS, and rheological properties were also measured. In hummus, both with and without garlic, chitosan added at 0.5% and 1% *w*/*w* significantly (*p* < 0.05) decreased LAB, aerobic bacteria, yeasts, and molds, and *Pseudomonas* spp., at 4 °C. However, at 10 °C, adding chitosan at 1% *w*/*w* significantly reduced only aerobic bacteria (2.2 log cfu/g) and *Pseudomonas* spp. (1.0 log cfu/g). The pH values (regardless of treatment) decreased upon storage. The addition of garlic or chitosan did not significantly affect the lightness (L*) or yellowness (b*). However, garlic, regardless of chitosan concentration, notably reduced lipid oxidation (0.8–1.4 MDA Eq/kg of sample) and had a greater impact on the sensory properties compared to chitosan. The results of this study will encourage producers to produce hummus that has a better flavor due to garlic with enhanced microbial quality.

## 1. Introduction

Chickpeas were one of the earliest cultivated legumes, with possible origins in the Levant region (Eastern Mediterranean) and Egypt [1]. Chickpeas are highly nutritious and considered a good and cheap source of dietary proteins. They are also a good source of polyunsaturated fatty acids, dietary fiber, prebiotic carbohydrates, vitamins, minerals, and polyphenols [1]. Therefore, chickpeas can be a healthy food choice for vegetarians and are a sustainable option compared to meat [1]. Hummus is the Arabic term for chickpea beans as well as chickpea-based dip. The traditional hummus dip is composed of three main ingredients: chickpeas, raw tahini (sesame paste), and lemon juice. To add variety, it is often seasoned with garlic, salt, pepper, olive oil and sumac [2,3].

Hummus is a ready-to-eat food product that is consumed directly without any further processing or additive treatments [4]. The only antimicrobial treatment hummus undergoes during preparation in foodservice establishments is boiling of chickpeas. As the water activity (a_w_) and pH values of hummus are approximately 0.98 and 6.5, respectively, it can provide a favorable environment for the growth of a variety of microorganisms if not properly treated [2,5]. Hummus may become contaminated with pathogenic bacteria, including *Salmonella enterica*, *Staphylococcus aureus*, *Escherichia coli* O157:H7, and *Listeria monocytogenes* [5]. In addition, the growth of certain microorganisms, such as lactic acid bacteria (LAB), *Pseudomonas* (PS) spp., and yeasts and molds (Y & M), can result in early hummus spoilage [6]. The aerobic plate count, LAB, and Y & M in hummus samples sold in Jordan were 5.8, 5.3, and 3 log_10_ CFU/g, respectively [7]. Controlling spoilage microorganisms in hummus is important in extending its shelf life. A study conducted on falafel paste made from chickpeas found *Salmonella* spp. could increase by up to 2 log_10_ CFU/g within 14 days at 10 °C [8].

Previous studies have evaluated the safety of hummus using different preservatives and preservation methods against pathogenic microorganisms [4,5,6,9,10,11,12]. Chitosan and garlic decreased *L. monocytogenes*, *E. coli* O157:H7, and *Salmonella* spp. by 1–2 log_10_ CFU/g in hummus stored at different temperatures [5]. Similarly, citric acid and garlic extract decreased *Salmonella enterica* and *L. monocytogenes* in hummus by an average of 3 log_10_ CFU/g [6]. Garlic exhibits this antimicrobial effect by disrupting the cell membrane, and this causes cell death [13]. On the other hand, chitosan exhibits its antimicrobial effect by changing the pH or the charge/permeability of the membrane. The mode of action depends on multiple factors like type of microorganism, concentration, the pH, and the surrounding environment [14].

However, limited information exists on their antimicrobial effects against spoilage-causing microorganisms.

Hummus can be classified as an emulsion, and lipid oxidation is a major concern in emulsions [15]. It leads to the degradation of lipids, which can alter the flavor, aroma, and taste of food products. Specifically, lipid oxidation results in the formation of rancid or off-flavors, such as fishy or cardboard-like tastes, due to the breakdown of unsaturated fatty acids [16]. In hummus, the primary lipids involved are the unsaturated fatty acids found in ingredients such as tahini. This process can negatively affect the overall sensory qualities and shelf life of hummus.

In a study conducted previously, the addition of garlic in raw ground beef decreased lipid oxidation significantly [17]. However, garlic can change color based on storage time and temperature of treatment. In one study it was observed that storage under room temperatures resulted in de-greening while storage at refrigeration temperatures increased greening [18].

On the other hand, although chitosan exhibits antimicrobial properties, it also has a thickening effect in emulsions [19].

Hummus dip is commonly prepared in batches and refrigerated until consumer use. A malfunctioning refrigerator or the repeated opening/closing of refrigerator doors can lead to an increase in temperature (to 10 °C or more). In addition, hummus is often stored at room temperature for extended periods during use in homes and while being served at restaurants, where it can be included on the buffet table. Such temperature abuse can stimulate the proliferation of spoilage microorganisms and shorten hummus shelf life by rendering it unsuitable for consumption.

Keeping the antimicrobial properties of garlic and chitosan and their capacity to change rheological properties, color, and oxidative stress, this study incorporated these two ingredients in hummus. The aims of the study were to better understand the role of garlic and chitosan on (i) spoilage-causing microorganisms (upon storage under refrigerative and temperature-abuse conditions), (ii) pH, (iii) color and lipid oxidation, (iv) textural properties, and (v) rheological properties in hummus.

## 2. Materials and Methods

### 2.1. Sample Preparation

Hummus was freshly prepared [5]. Chickpeas were soaked in water for 8 h. This was followed by boiling for 2 h in water containing 0.83% (*w*/*w*) sodium bicarbonate. The boiled chickpeas were then spread on a large stainless-steel tray for 90 min at 4 °C. To prepare the hummus dip, the cooled chickpeas were mashed using a sterilized mixer at a speed of 6000 rpm for 2 min, and then the tahini pulp was added. Six batches of hummus were prepared; each batch was 2.1 kg and consisted of 1050 g cooked, drained chickpeas (which accounted for 50.0% of the total batch weight), 690 g water (32.9%), 345 g tahini (16.4%), 15 g citric acid (0.7%), and 12.6 g NaCl (0.6%). Minced fresh garlic at 1% (*w*/*w*) was added only to three hummus batches. All ingredients were obtained from the local market.

In chitosan–hummus treatments, low molecular weight chitosan (<100 kDa) with a deacetylation degree of 75–85% (Sigma-Aldrich, St. Louis, MO, USA) was used to prepare the chitosan solution. Garlic was added to hummus with or without 0.5% or 1% chitosan. Chitosan concentrations were chosen based on a previously published study [5]. In order to yield a final concentration of 0.5% and 1.0% chitosan in hummus, 2.7 g acetic acid (0.33%) plus 10.5 g (1.52%) or 21 g (3.04%) chitosan were mixed with water to develop a 690 g emulsion.

The hummus samples were then stored for 8 intervals at each of three temperatures (0, 4, 8, 12, 16, 20, 24, and 28 d at 4 °C; 0, 3, 6, 9, 12, 15, 18, and 21 d at 10 °C; and 0, 1, 2, 3, 4, 5, 6, and 7 d at 25 °C).

### 2.2. Bacterial Enumeration

After storage, 10 g of hummus was aseptically transferred to sterile stomacher bags containing 90 mL of peptone water (Himedia, Mumbai, India). Homogenization of the samples was conducted using a stomacher (Easy Mix, AES Laboratoire, Bruz, France) for 2 min [20,21]. Then, 0.1 or 1 mL of appropriate decimal dilutions was spread-plated in duplicate. Lactic acid bacteria were recovered on de Man Rogosa Sharpe agar (MRS), which was incubated anaerobically at 25 °C for 5 d. Aerobic plate count (APC) numbers were recovered on plate count agar (PCA) incubated aerobically at 30 °C for 3 d. Yeast and mold numbers were estimated on potato dextrose agar (PDA) incubated aerobically at 25 °C for 5 d, and *Pseudomonas* (PS) were recovered after plating on *Pseudomonas* Agar Base supplemented with *Pseudomonas* CFC incubated aerobically at 25 °C for 2 d (Sigma-Aldrich, Hamburg, Germany). Results were expressed in log_10_ CFU/g.

### 2.3. PH Measurement

pH was measured using a pH meter (OHAUS Starter 3100, Ohaus Corporation, Parsippany, NJ, USA) under all the storage conditions at the three temperatures (4, 10 and 25 °C, respectively).

### 2.4. Color Measurement

The color was measured at room temperature using a Hunterlab ColorFlex EZ Colorimeter (HunterLab, Reston, VA, USA). Results were interpreted with reference to the CEILAB system as L* (black and white representing 0 and 100, respectively), a* (red (>0) to green (<0) color range), and b* (yellow (>0) to blue (<0) color range) [22].

### 2.5. Thiobarbituric Acid-Reactive Substances (TBARS) Test

The test was performed to measure lipid oxidation [23]. A thiobarbituric acid (TBA) solution was prepared by mixing 15% of trichloroacetic acid (TCA), 0.375% of TBA, 2% of 0.25 N HCl, and deionized water. The TBA solution and hummus sample were mixed in a 5:1 ratio and heated in boiling water for 10 min until pink. This was followed by cooling the mixture at room temperature and centrifugation (10,000 rpm for 15 min). The absorbance was measured at 532 nm using a UV spectrophotometer (Shimadzu UV-1800, Kyoto, Japan). Water was used as a control. The standard curve was prepared using 1,1,3,3-tetramethoxypropane (MAD) at a 0 to 10 ppm concentration range. The TBARS results were expressed as mg of MAD equivalents/kg of sample.

### 2.6. Rheological Investigations

#### 2.6.1. Texture Profile Analysis (TPA)

The TPA was conducted using a texture analyzer CT3 (Brookfield Ametek, Harlow, UK). A cylindrical probe of 25 mm, a load cell of 4.5 kg, and a trigger force of 6.8 g at a 0.5 mm/s test speed for a 10 mm target distance in a 100 g sample were used for this purpose [24].

#### 2.6.2. Rheological Properties

About 3 g of hummus samples were analyzed using a rheometer (Discovery Hybrid HR-2, TA Instruments, New Castle, DE, USA). The parameters were set at a 500 μm gap and a plate-controlled temperature of 25 ± 0.1 °C. The parallel plate was 40 mm in diameter. All data were analyzed using TRIOS 5.2 software (TA Instruments, DE, USA).

#### 2.6.3. Strain and Frequency Sweep Tests

A strain sweep test and frequency sweep test were used to analyze the linear viscoelastic region. A strain range of 0.01–10% was used at a constant frequency of 1.0 Hz. A linearity of 0.3% was observed and thereby used for later tests. Similarly, for the frequency sweep test, a frequency range of 0.1 and 50 Hz and a constant strain of 0.3% within the linear viscoelastic region were utilized.

#### 2.6.4. Time-Dependent Behavior

The thixotropic behavior of the hummus samples was measured with low and high shearing conditions as described by [25] to determine the structural deformation and recovery. The storage (G′) and loss (G″) moduli were measured using an oscillation–time test at a constant frequency of 1.0 Hz over three time segments: firstly (200 s, 0.3% strain), secondly (60 s, 50% strain), and thirdly (400 s, 0.3% strain).

### 2.7. Statistical Analysis

Statistical analysis was carried out using IBM Statistical Package for Social Sciences (SPSS, version 25.0) (Chicago, IL, USA). One-way ANOVA was used to examine the impact of different variables, such as chitosan concentration, storage time, and their interaction on the viability (log_10_ CFU/g) of the tested microorganisms, color, and lipid oxidation. A *t*-test was used to examine the difference in effects between the presence and absence of garlic. Furthermore, another one-way ANOVA was conducted to evaluate the influence of chitosan concentration and garlic presence on the color and lipid oxidation of hummus. A *p*-value of less than 0.05 was considered to be statistically significant. All experiments were conducted in triplicate.

## 3. Results and Discussion

### 3.1. Effect of Garlic and Chitosan on Spoilage-Causing Microorganisms

As a ready-to-eat food product, hummus is very susceptible to microbial growth due to its high a_w_ [2]. Yamani and Al-Dababseh (1994) collected samples from different restaurants in Jordan and reported finding large numbers of LAB, especially in samples collected during the summer [3]. Artificial preservatives and stabilizers can be used to extend the shelf life of hummus; however, consumers usually prefer natural alternatives.

In this study, natural antimicrobials, namely garlic and chitosan, have been used. The data are presented in Table 1, Table 2, Table 3 and Table 4. Fresh garlic is known for its antimicrobial and antioxidant action against spoilage bacteria [26]. In the current study, adding garlic to hummus followed by storage at 4, 10, and 25 °C resulted in a maximum decrease in APC of 0.5 (day 16 and 20), 1.3 (day 12), and 1.4 (day 7) log_10_ CFU/g, respectively. Similarly, garlic decreased LAB by a maximum of 1.0 (day 24), 0.7 (day 3), and 0.8 (day 5) log_10_ CFU/g, respectively. Meanwhile, the maximum decreases in Y & M at 4, 10, and 25 °C upon garlic addition were 2.5 (day 28), 1.1 (day 21), and 1.4 (day 6) log_10_ CFU/g, respectively. Furthermore, incorporation of garlic into hummus decreased PS by a maximum of 1.3 (day 24), 1.0 (day 18), and 1.2 (days 3 and 5) log_10_ CFU/g, respectively. There was a trend toward greater inhibitory action of garlic on LAB, Y & M, and PS observed at 4 °C than at 10 or 25 °C. The addition of 30 g/kg fresh garlic or 9 g/kg garlic powder significantly reduced the APC and extended the shelf life of chicken sausage to 21 d [27]. Olive oil added to chickpea spread was observed to better control total viable counts as compared to sunflower oil after storage for a day at 4 °C [28]. Osaili et al. (2022) reported that adding chitosan and garlic to hummus dip reduced the numbers of *Salmonella* spp., *E. coli* O157:H7, and *L. monocytogenes* [5]. In shrimp, the combined effect of garlic oil and chitosan coating resulted in a reduction of APC more than in shrimp coated with chitosan alone [29]. A previously published study suggested that the antimicrobial properties of garlic and chitosan were more effective at lower temperatures against *Salmonella enterica* [30].

Overall, in comparison with garlic, chitosan had a greater antimicrobial effect at all temperatures on the microorganisms examined. Chitosan has previously been reported to exhibit substantial antimicrobial activity against a variety of pathogenic and spoilage microorganisms, particularly at pH values lower than 6.0 [31,32]. The exact mechanism of chitosan’s antibacterial activity is yet to be fully understood; however, it is suggested that chitosan works by binding to the cell wall of pathogenic bacteria, increasing membrane permeability, which leads to disruption of cell integrity. It also results in cell death by preventing DNA replication [33,34]. In the current study, chitosan at 1% was observed to have a greater antimicrobial effect than at 0.5%. Chitosan individually decreased (*p* < 0.05) APC, LAB, Y & M, and PS by a maximum of 3.2 log_10_ CFU/g (1% chitosan, day 21, 10 °C, hummus without garlic); by 1.8 log_10_ CFU/g (1% chitosan, day 21, 10 °C, hummus with garlic); by 3.2 log_10_ CFU/g (1% chitosan, day 28, 4 °C, hummus with garlic); and by 3.2 log_10_ CFU/g (1% chitosan, day 28, 4 °C, hummus with garlic), respectively. In previous work, it was reported that adding chitosan to hummus dip reduced the numbers of *Salmonella* spp., *E. coli* O157:H7, and *L. monocytogenes* [5]. The addition of 0.5% chitosan and 1% garlic resulted in hummus of overall fair acceptability [5]. Chitosan showed an antibacterial effect on PS in refrigerated Pacific oysters and increased their shelf life from 8–9 d to 14–15 d [35]. Latou et al. (2014) also observed that dipping chicken fillets in a chitosan solution (1 g/100 mL) extended the shelf life of the product by 6 d [36]. Tokatl and Demirdöven (2020) reported that chitosan coatings were effective in inhibiting Y & M at 4 °C for 25 d [37]. Similarly, Sucharitha et al. (2018) reported that tomatoes coated with chitosan had lower total plate count and Y & M numbers compared to the control [38]. Moreover, chitosan coating of fresh-cut broccoli had a significant (*p* < 0.05) bactericidal effect on psychrotrophic and mesophilic aerobes, causing reductions of 1.5–2.5 log_10_ CFU/g, while LAB had relatively low numbers (2.5–4.0 log_10_ CFU/g) during the entire storage period [39]. Zheng and Zhu (2003) reported that the effect of differences in molecular weight of chitosan affected the antimicrobial susceptibility of Gram-positive and -negative bacteria differently, and this appeared to be related to the ability of chitosan to access and penetrate the cell membrane [40]. In other work, Chung et al. (2004) observed that increased bacterial surface electronegativity was correlated with increased surface absorption of chitosan and greater bacterial inhibition [41]. The antimicrobial action of chitosan is influenced by several factors, some intrinsic (e.g., type of chitosan, its molecular weight, or degree of polymerization) and some extrinsic (e.g., target microorganism, environmental conditions, pH, temperature, and the presence of other components) [42,43].

The combination of garlic and 1% chitosan decreased APC up to 0.50 log_10_ CFU/g (day 4 and 12), 3.3 log_10_ CFU/g (day 21), and 2.9 log_10_ CFU/g (day 7) at 4, 10, and 25 °C, respectively (*p* < 0.05). Meanwhile, the decrease in LAB at the same temperatures was 1.7 log_10_ CFU/g (day 24 and 28), 2.0 log_10_ CFU/g (day 12), and 2.3 log_10_ CFU/g (day 6), respectively. The 1% chitosan and garlic mixture also decreased Y & M by up to 4.3 log_10_ CFU/g (day 28), 2.2 log_10_ CFU/g (day 21), and 2.6 log_10_ CFU/g (day 7) at 4, 10, and 25 °C, respectively. Decreases in PS by a maximum of 3.5 log_10_ CFU/g (day 28), 2.8 log_10_ CFU/g (day 21), and 3.1 log_10_ CFU/g (day 7), respectively, were also recorded under the same conditions. It was expected that the overall mixture of chitosan and garlic would result in a synergistic action against microbial populations; however, this was not the case. It is possible that the antimicrobial effect of chitosan was affected by the various other bioactive compounds inherent to garlic.

### 3.2. Effect of Garlic and Chitosan on pH of Hummus

The pH of hummus (regardless of treatment) decreased upon storage (Table 5). Overall, the addition of garlic to hummus decreased the pH transformation at the end of the storage period. Towards the end of the storage period, the average change in pH in hummus stored without garlic at 4, 10, and 25 °C was by 0.65, 1.01, and 1.39, respectively (*p* < 0.05). Meanwhile, in hummus with garlic, the mean change in pH towards the end of the storage period was by 0.51, 0.61, and 1.07, respectively. Increasing the concentration of chitosan from 0 to 1% increased the pH at 4 and 10 °C. The decrease in pH observed towards the end of the storage period (observed under all storage conditions) could be due to the activity of spoilage microbiota or the naturally occurring microbiota [44]. LAB produce various organic acids like lactates, acetates, and butyrate upon their growth [45].

### 3.3. Effect of Garlic and Chitosan on the Color of Hummus

Neither the addition of garlic nor chitosan in both concentrations (0.5 or 1.0%) significantly changed lightness (L*) (Table 6). The addition of 1% chitosan (without garlic) significantly (*p* < 0.001) decreased A*. The values for B* remained consistent around a value of 19 regardless of the type and concentration of additives. Chitosan is usually used as a coating agent in foods. However, in one study it was used as a clarifying agent in pomegranate juice [46]. It was observed that chitosan did not majorly change the L*, A*, or B* values, which is in accordance with the results of this study. On the other hand, garlic changes its color upon storage [47]. No significant change in color after its addition was expected as the measurements were performed immediately without any storage.

### 3.4. Effect of Garlic and Chitosan on TBARS of Hummus

The addition of garlic to hummus, regardless of the chitosan concentration, decreased lipid oxidation significantly (*p* < 0.001) (Table 7). This could be attributed to the flavonoids and other organosulfur compounds that are inherent to garlic [48]. On the other hand, it was observed that chitosan increased the TBARs significantly at a 0.5% concentration without garlic. This could be because TBARS can react with substances other than lipid oxidation products, such as sugars, amino acids, and oxidized proteins, and this may alter the results obtained [49].

### 3.5. Effect of Garlic and Chitosan on the Rheological Properties of Hummus

**Figure 1:**
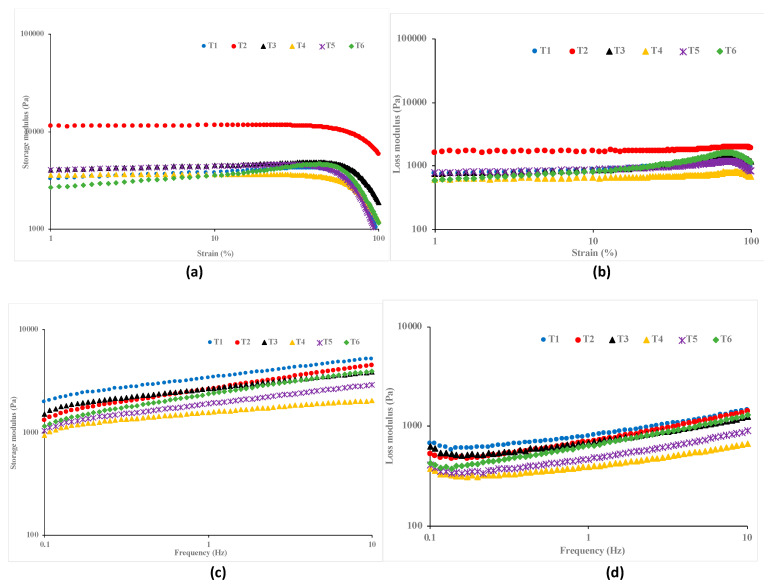
Rheological linear test of hummus (T1: hummus + No Garlic + No chitosan (control), T2: hummus + No Garlic + 0.5% chitosan, T3: hummus + No Garlic + 1.0% chitosan, T4: hummus + 1% Garlic + No chitosan, T5: hummus + 1% Garlic + 0.5% chitosan, T6: hummus + 1% Garlic + 1.0% chitosan). (**a**) Storage modulus, strain (%); (**b**) Loss modulus, strain (%); (**c**): Storage modulus, Frequency (Hz); (**d**) Loss modulus, Frequency (Hz).

Rheological investigations are crucial in comprehending the flow behavior of food materials, which is necessary for evaluating the potential variations in the structural, textural, and compositional changes that might arise during food preparation [50]. One such test is the strain sweep test, which gives information about the linear viscoelastic regions (LVR) of the sample. The linearity test of the garlic- and chitosan-treated hummus samples was evaluated in the strain range of 1–100%, and results are shown in Figure 1a,b in the form of storage (G′) and loss (G″) moduli. As shown in Figure 1, the LVR of the hummus samples varies with the treatment type and moduli. Specifically, the highest strains to maintain a linearity system in the samples were 10% and about 80% for the G′ (Figure 1a) and G″ (Figure 1b) modulus, respectively. Additionally, the G′ is dominant over the G″ in the LVR, confirming a more elastic property and lower viscosity of the hummus samples. Similar findings have been reported in previous studies [24,50,51].

**Figure 2:**
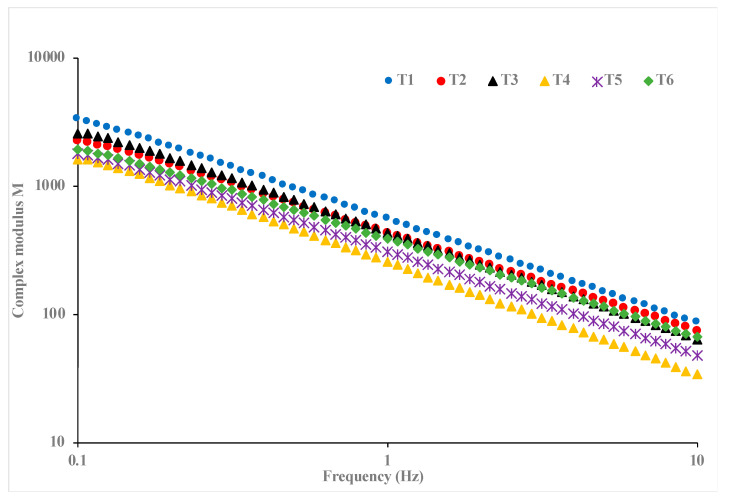
Viscoelastic properties of hummus (T1: hummus + No Garlic + No chitosan (control), T2: hummus + No Garlic + 0.5% chitosan, T3: hummus + No Garlic + 1.0% chitosan, T4: hummus + 1% Garlic + No chitosan, T5: hummus + 1% Garlic + 0.5% chitosan, T6: hummus + 1% Garlic + 1.0% chitosan).

The frequency sweep test of the garlic and chitosan-treated hummus samples was conducted over a frequency range of 1–10 Hz, and the results are presented in the figure in the form of storage (G′), loss (G″), and complex moduli. Overall, the results showed that G′ and G″ values increase with an increase in the applied frequency with G′ values predominantly higher than G″ for all samples, which is consistent with earlier studies on hummus samples [24,51,52]. The observed G′ > G″ trend is indicative that the hummus samples, irrespective of the treatments, have high elasticity and low viscosity properties, although variations exist in their viscoelastic behavior. Specifically, hummus with no garlic and chitosan and hummus with 1% garlic and no chitosan exhibited the highest and least viscoelastic properties, respectively, evidenced by the consistent high and low values of G′ and G″ throughout the applied frequency range. These variations in viscoelastic properties may be attributed to the influence of the treatment on the chickpeas and sesame seed oil protein network structure. Similar findings were observed in chitosan-treated whey protein [53]. Additionally, the complex viscosity (Figure 2) decreases with an increase in the applied frequency, with hummus without garlic and chitosan and hummus with 1% garlic and no chitosan having the least and highest complex modulus, respectively. This phenomenon indicates a shear-thinning behavior of all the samples [50].

**Figure 3:**
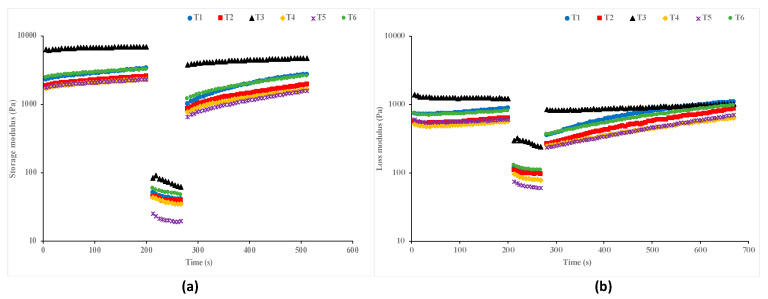
Thixotropic behavior of hummus [(**a**): G′ and (**b**): G″] (T1: hummus + No Garlic + No chitosan (control), T2: hummus + No Garlic + 0.5% chitosan, T3: hummus + No Garlic + 1.0% chitosan, T4: hummus + 1% Garlic + No chitosan, T5: hummus + 1% Garlic + 0.5% chitosan, T6: hummus + 1% Garlic + 1.0% chitosan).

The time sweep test gives information about material restructuring and reformation following the application of high shear stress. The time sweep test of the garlic and chitosan-treated hummus samples was evaluated with a three-step shear stress test of very low (0.8%), high (50%), and very low (0.8%) strain, and the results are presented in Figure 3a,b. Overall, the applied high shear stress disrupted the hummus structure. All the samples exhibited full regeneration to almost the initial values of their storage (G′) (Figure 3a) and loss (G″) (Figure 3b) moduli, indicating the time-independent behavior of the hummus samples. Particularly, at 0.8% strain, the hummus samples showed an aggregate of G′ > G″ stability for about 200 s, indicating a higher elastic property of the samples, after which G″ was dominant over G′ for about 100 s, which is indicative of the gel-sol-like transition of all the hummus samples. Following deformation, the G′ slowly increased from 300 s and became dominant over G″ up to 600 s. Similar findings have been reported by several studies [25,50]. The difference in the restructuring and reformation may be attributed to the effect of the chitosan and garlic treatment on the network structure of the hummus component [54].

The textural characteristics, including hardness, adhesiveness, and stringiness, of garlic and chitosan-treated hummus samples at varying concentrations are outlined in Table 8. Hummus textural properties are notably affected by factors such as particle aggregation, protein constituents, and quantities, which contribute to forming a fragile gel network structure [24,50,55]. As seen in Table 8, the hardness, adhesiveness, and stringiness of the evaluated hummus samples vary with the type and concentration of the garlic and chitosan. Specifically, the hardness values of the hummus samples ranged between 96.9 to a peak value of 108, corresponding to the control (without garlic and chitosan) and hummus containing a combination of 1% of garlic and 1% chitosan, respectively. This is indicative of the concentration-dependent effect of garlic and chitosan treatment on the structural properties of the hummus samples to produce a stronger gel network. Additionally, there was a depreciation in the hardness value when the hummus sample was treated with only 1% of garlic without chitosan. Hence, we believe that the appreciated hardness in T6 was the synergistic effect of both garlic and chitosan. Our findings are consistent with the results of a previous study conducted on chitosan-treated rice but in contrast to who reported the lowest hardness in lentil-based tofu treated with 1% chitosan [56,57]. The adhesive properties of the hummus samples showed a decreasing and increasing trend with the treatment type. Notably, there is no significant difference (*p* < 0.05) in the adhesiveness properties of the treatment groups and the control, which aligns with previous results from different studies [56,57,58]. Similarly, the hummus samples showed an increasing and decreasing trend in the observed stringiness values, which is significantly different (*p* < 0.05) from the control group. The least (8.4 mm) stringiness value was exhibited by the control group, while the highest value (9.3 mm) was observed in the hummus sample treated with 1% garlic, which is not significantly different from the sample treated with 1% garlic and 0.5% chitosan. Hence, we believe the garlic had more influence on the stringiness property of the hummus compared to chitosan. Similar findings were found in chitosan-treated lentil-based tofu [56] and garlic polysaccharide-treated dough [59].

## 4. Conclusions

This study demonstrates the potential of incorporating garlic and chitosan into hummus to enhance its quality. Garlic is known for its antioxidant properties, which may contribute to overall health benefits. Chitosan, with its proven antimicrobial and preservative effects, can help extend the shelf life of hummus while maintaining its sensory appeal. Both ingredients offer promising applications in developing innovative food products that align with consumer demand for natural, health-conscious options. The safety of chitosan as a food additive is well-established, with regulatory approvals in various regions. This research lays the groundwork for further exploration of these ingredients in food formulations to improve product quality and safety. Future studies could assess the long-term effects of garlic and chitosan in food products and their broader health benefits.

## Figures and Tables

**Table 1:** Population changes of lactic acid bacteria in hummus with or without garlic at different chitosan concentrations stored for 28 d at 4 °C, 21 d at 10 °C, or 7 d at 25 °C.

**Temperature**	**Lactic Acid Bacteria (log_10_ CFU/g)**										
		**Garlic**	**Chitosan**	**Days**							
				0	4	8	12	16	20	24	28
	4 °C	No	0	0.0	0.03 ± 0.05 ^Ac^	−0.01 ± 0.03 ^Ac^	0.23 ± 0.04 ^Ab^	0.47 ± 0.07 ^Aab^	0.39 ± 0.02 ^Aa^	0.88 ± 0.05 ^Aa^	0.94 ± 0.01 ^Aa^
		No	0.5	0.0	−0.06 ± 0.01 ^Aa^	−0.06 ± 0.04 ^Aa^	−0.12 ± 0.04 ^Ba^	−0.15 ± 0.07 ^Ba^	−0.06 ± 0.07 ^Ba^	−0.05 ± 0.03 ^Ba^	−0.05 ± 0.01 ^Ba^
		No	1	0.0	0.00 ± 0.01 ^Ab^	−0.15 ± 0.02 ^Aa^	−0.15 ± 0.02 ^Ba^	−0.15 ± 0.03 ^Ba^	−0.07 ± 0.00 ^Ba^	−0.15 ± 0.01 ^Ca^	−0.15 ± 0.01 ^Ca^
		Yes	0	0.0	0.01 ± 0.05 ^Ac^	0.10 ± 0.00 ^Ac^	0.30 ± 0.01 ^Ac^	0.51 ± 0.05 ^Ab^	0.49 ± 0.03 ^Ab^	0.54 ± 0.02 ^Aa^	0.66 ± 0.00 ^Aa^
		Yes	0.5	0.0	−0.10 ± 0.03 ^Aa^	−0.02 ± 0.04 ^Aa^	−0.01 ± 0.00 ^Ba^	0.00 ± 0.02 ^Ba^	−0.10 ± 0.01 ^Ba^	−0.02 ± 0.05 ^Ba^	−0.01 ± 0.00 ^Ba^
		Yes	1	0.0	0.00 ± 0.12 ^Aa^	0.00 ± 0.03 ^Aa^	−0.10 ± 0.03 ^Ba^	−0.10 ± 0.00 ^Ba^	−0.10 ± 0.04 ^Ba^	−0.10 ± 0.20 ^Ba^	−0.10 ± 0.11 ^Ba^
				0	3	6	9	12	15	18	21
	10 °C	No	0	0.0	0.28 ± 0.04 ^Ad^	0.50 ± 0.04 ^Ac^	0.69 ± 0.18 ^Abc^	1.75 ± 0.16 ^Aa^	1.80 ± 0.49 ^Aa^	2.10 ± 0.02 ^Aa^*	2.15 ± 0.21 ^Aa^*
		No	0.5	0.0	0.00 ± 0.00 ^Ad^	0.10 ± 0.09 ^Ac^	0.30 ± 0.06 ^Bc^	0.81 ± 0.37 ^Bab^	1.00 ± 0.06 ^Ba^*	1.20 ± 0.07 ^Ba^	1.32 ± 0.04 ^Ba^
		No	1	0.0	0.00 ± 0.21 ^Ad^	0.10 ± 0.04 ^Ac^	0.01 ± 0.12 ^Bc^	0.20 ± 0.46 ^Cc^	0.30 ± 0.20 ^Cbc^	0.51 ± 0.28 ^Ca^	0.68 ± 0.05 ^Ca^
		Yes	0	0.0	0.30 ± 0.06 ^Ad^	0.54 ± 0.11 ^Ad^	0.78 ± 0.18 ^Ac^	1.80 ± 0.91 ^Abc^	2.00 ± 0.49 ^Ab^	2.37 ± 0.02 ^Aab^	2.76 ± 0.02 ^Aa^
		Yes	0.5	0.0	0.00 ± 0.12 ^Ad^	0.24 ± 0.09 ^Bcd^	0.44 ± 0.25 ^ABc^	0.96 ± 0.77 ^ABb^	1.44 ± 0.01 ^Aba^	1.35 ± 0.22 ^Aa^	1.52 ± 0.04 ^Ba^
		Yes	1	0.0	0.00 ± 0.49 ^Ac^	0.18 ± 0.02 ^Bbc^	0.24 ± 0.02 ^Bbc^	0.48 ± 0.04 ^Babc^	0.69 ± 0.21 ^Bab^	0.95 ± 0.05 ^Ba^	0.93 ± 0.10 ^Ca^
				0	1	2	3	4	5	6	7
	25 °C	No	0	0.0	0.34 ± 0.12 ^Ac^	0.35 ± 0.00 ^Ac^	1.2 ± 0.12 ^Ab^*	1.90 ± 0.04 ^Ab^	2.53 ± 0.01 ^Aa^	2.99 ± 0.00 ^Aa^*	3.00 ± 0.05 ^Aa^*
		No	0.5	0.0	0.20 ± 0.12 ^Ac^	0.20 ± 0.09 ^Ac^	0.35 ± 0.06 ^Bc^	1.26 ± 0.06 ^Bb^	1.30 ± 0.01 ^Bb^	2.0 ± 0.07 ^Ba^	2.10 ± 0.04 ^Ba^
		No	1	0.0	0.10 ± 0.17 ^Ac^	0.15 ± 0.04 ^Ac^	0.19 ± 0.01 ^Bc^	0.65 ± 0.21 ^Cb^	0.95 ± 0.00 ^Ca^	1.00 ± 0.46 ^Ca^	1.00 ± 0.06 ^Ca^
		Yes	0	0.0	0.43 ± 0.12 ^Ad^	0.54 ± 0.00 ^Ad^	1.69 ± 0.20 ^Ac^	2.00 ± 0.47 ^Aab^	2.45 ± 0.01 ^Aa^	3.00 ± 0.66 ^Aa^	3.21 ± 0.05 ^Aa^
		Yes	0.5	0.0	0.26 ± 0.09 ^Ae^	0.24 ± 0.00 ^Be^	0.44 ± 0.00 ^Bd^	1.206 ± 0.21 ^Bc^	1.39 ± 0.09 ^Bc^	2.35 ± 0.10 ^Bb^	2.52 ± 0.10 ^Ba^
		Yes	1	0.0	0.24 ± 0.11 ^Ab^	0.15 ± 0.11 ^Bb^	0.24 ± 0.11 ^Bb^	0.78 ± 0.02 ^Cc^	1.16 ± 0.04 ^Bb^	1.33 ± 0.05 ^Cb^	1.63 ± 0.10 ^Ca^

Results are mean values of three replications, expressed as log_10_ CFU/g differences compared to the initial time (time 0). Mean values at time 0: 2.28 (with garlic), 2.96 (without garlic). Numbers in the same column with the same uppercase letters are NOT significantly different (*p* > 0.05). Numbers in the same row with the same lowercase letters are NOT significantly different (*p* > 0.05). * Indicates significant difference in effects between the presence and absence of garlic (*p* < 0.05).

**Table 2:** Population changes of aerobic plate counts in hummus with or without garlic at different chitosan concentrations stored for 28 d at 4 °C, 21 d at 10 °C, or 7 d at 25 °C.

**Temperature**	**Aerobic Plate Counts (log_10_ CFU/g)**										
		**Garlic**	**Chitosan**	**Days**							
				0	4	8	12	16	20	24	28
	4 °C	No	0	0.0	0.11 ± 0.23 ^Aa^	0.10 ± 0.03 ^Aa^	0.10 ± 0.00 ^Aa^	0.20 ± 0.07 ^Aa^	0.30 ± 0.00 ^Aa^	−0.10 ± 0.34 ^Ab^	0.15 ± 0.00 ^Aa^
		No	0.5	0.0	0.00 ± 0.15 ^Aba^	0.10 ± 0.11 ^Ba^	0.00 ± 0.03 ^Ba^	0.00 ± 0.03 ^Ba^	−0.10 ± 0.05 ^Ba^	0.10 ± 0.06 ^Ba^	−0.1 ± 0.04 ^Ba^
		No	1	0.0	−0.27 ± 0.17 ^Ba^	−0.15 ± 0.00 ^Ca^	−0.25 ± 0.08 ^Ba^	−0.32 ± 0.18 ^Ba^	−0.39 ± 0.09 ^Ca^	−0.13 ± 0.03 ^Ca^	−0.13 ± 0.03 ^Ca^
		Yes	0	0.0	0.00 ± 0.21 ^Aa^	0.10 ± 0.03 ^Aa^	0.00 ± 0.00 ^Aa^	−0.20 ± 0.07 ^Aa^	−0.10 ± 0.02 ^Aa^	0.00 ± 0.04 ^Aa^	0.00 ± 0.02 ^Aa^
		Yes	0.5	0.0	−0.18 ± 0.01 ^Ba^	−0.10 ± 0.21 ^Ba^	−0.20 ± 0.13 ^Ba^	−0.20 ± 0.43 ^Ba^	−0.10 ± 0.25 ^Ba^	0.00 ± 0.01 ^Ba^	−0.20 ± 0.04 ^Ba^
		Yes	1	0.0	−0.32 ± 0.11 ^Ba^	−0.20 ± 0.02 ^Ca^	−0.26 ± 0.01 ^Ba^	−0.10 ± 0.18 ^Ba^	0.00 ± 0.19 ^Ca^	−0.19 ± 0.00 ^Ca^	−0.19 ± 0.03 ^Ca^
				0	3	6	9	12	15	18	21
	10 °C	No	0	0.0	0.65 ± 0.03 ^Ad^	1.49 ± 0.45 ^Ac^*	2.35 ± 0.28 ^Abc^*	2.71 ± 0.14 ^Ab^*	2.85 ± 0.03 ^Ab^*	2.96 ± 0.27 ^Aab^*	3.15 ± 0.00 ^Aa^*
		No	0.5	0.0	0.15 ± 0.00 ^Bc^	0.01 ± 0.17 ^Bc^	0.00 ± 0.24 ^Bc^	0.36 ± 0.27 ^Bb^	0.54 ± 0.01 ^Ba^	0.59 ± 0.06 ^Ba^	0.66 ± 0.02 ^Ba^
		No	1	0.0	−0.10 ± 0.09 ^Cb^	−0.20 ± 0.23 ^Bb^	−0.3 ± 0.06 ^Bb^	−0.28 ± 0.18 ^Cb^	0.00 ± 0.20 ^Ca^	0.01 ± 0.34 ^Ca^	0.00 ± 0.23 ^Ca^
		Yes	0	0.0	0.60 ± 0.01 ^Ad^	0.95 ± 0.02 ^Ad^	1.25 ± 0.02 ^Ac^	1.52 ± 0.11 ^Ac^	2.00 ± 0.09 ^Ab^	2.22 ± 0.08 ^Aa^	2.69 ± 0.32 ^Aa^
		Yes	0.5	0.0	0.00 ± 0.13 ^Bb^	0.01 ± 0.21 ^Bb^	0.00 ± 0.17 ^Bb^	0.00 ± 0.12 ^Bb^	0.20 ± 0.13 ^Ba^	0.01 ± 0.14 ^Ba^	0.51 ± 0.15 ^Ba^
		Yes	1	0.0	−0.33 ± 0.09 ^Ba^	−0.38 ± 0.14 ^Ca^	−0.41 ± 0.32 ^Ba^	−0.38 ± 0.03 ^Ba^	−0.01 ± 0.01 ^Ba^	−0.10 ± 0.20 ^Ba^	−0.01 ± 0.20 ^Ca^
				0	1	2	3	4	5	6	7
	25 °C	No	0	0.0	0.50 ± 0.11 ^Ae^	0.99 ± 0.71 ^Ad^	0.81 ± 0.06 ^Ad^	1.67 ± 0.00 ^Ac^*	1.98 ± 0.21 ^Ac^*	2.05 ± 0.20 ^Ab^*	2.98 ± 0.21 ^Aa^*
		No	0.5	0.0	0.02 ± 0.18 ^Bc^	−0.13 ± 0.03 ^Bc^	0.35 ± 0.12 ^Bb^	0.44 ± 0.10 ^Bab^	0.66 ± 0.01 ^Ba^	0.78 ± 0.08 ^Ba^	0.77 ± 0.04 ^Ba^
		No	1	0.0	−0.02 ± 0.03 ^Bb^	−0.15 ± 0.06 ^Bb^	0.04 ± 0.16 ^Cb^	0.06 ± 0.03 ^Cb^	0.24 ± 0.09 ^BCa^	0.24 ± 0.34 ^BCa^	0.32 ± 0.03 ^BCa^
		Yes	0	0.0	0.23 ± 0.02 ^Ad^	0.81 ± 0.12 ^Ac^	0.87 ± 0.12 ^Ac^	1.00 ± 0.54 ^Aab^	1.25 ± 0.40 ^Aa^	1.52 ± 0.01 ^Aa^	1.66 ± 0.02 ^Aa^
		Yes	0.5	0.0	0.02 ± 0.12 ^Bc^	−0.13 ± 0.09 ^Bc^	0.27 ± 0.25 ^ABb^	0.33 ± 0.11 ^Ba^	0.54 ± 0.01 ^Ba^	0.57 ± 0.22 ^Ba^	0.67 ± 0.04 ^Ba^
		Yes	1	0.0	−0.02 ± 0.04 ^Ba^	−0.20 ± 0.02 ^Ba^	0.00 ± 0.02 ^Ba^	0.00 ± 0.01 ^Ca^	0.10 ± 0.21 ^Ca^	0.12 ± 0.05 ^Ca^	0.21 ± 0.10 ^Ca^

Results are mean values of three replications, expressed as log_10_ CFU/g differences compared to the initial time (time 0). Mean values at time 0: 2.28 (with garlic), 2.96 (without garlic). Numbers in the same column with the same uppercase letters are NOT significantly different (*p* > 0.05). Numbers in the same row with the same lowercase letters are NOT significantly different (*p* > 0.05). * Indicates significant difference in effects between the presence and absence of garlic (*p* < 0.05).

**Table 3:** Population changes of yeasts and molds in hummus with or without garlic at different chitosan concentrations stored for 28 d at 4 °C, 21 d at 10 °C, or 7 d at 25 °C.

**Temperature**	**Yeast and Mold Counts (log_10_ CFU/g)**										
		**Garlic**	**Chitosan**	**Days**							
				0	4	8	12	16	20	24	28
	4 °C	No	0	0.0	0.30 ± 0.01 ^Aa^	0.30 ± 0.05 ^Aa^	0.40 ± 0.14 ^Aa^	0.20 ± 0.32 ^Aa^	0.30 ± 0.08 ^Aa^	0.40 ± 0.09 ^Aa^	0.60 ± 0.12 ^Aa^
		No	0.5	0.0	−0.31 ± 0.15 ^Ba^	−0.61 ± 0.04 ^Ba^	−0.026 ± 0.18 ^Ba^	−0.22 ± 0.03 ^Ba^	−0.44 ± 0.06 ^Ba^	−0.31 ± 0.29 ^Ba^	−0.24 ± 0.33 ^Ba^
		No	1	0.0	−0.67 ± 0.15 ^Ba^	−0.95 ± 0.21 ^BCb^	−1.00 ± 0.27 ^Cb^	−1.00 ± 0.03 ^Cb^	−1.06 ± 0.18 ^Cb^*	−1.06 ± 0.24 ^Cb^*	−1.15 ± 0.12 ^Cb^*
		Yes	0	0.0	0.29 ± 0.14 ^Aa^	0.20 ± 0.00 ^Aa^	0.10 ± 0.12 ^Aa^	0.20 ± 0.07 ^Aa^	0.20 ± 0.03 ^Aa^	0.10 ± 0.28 ^Aa^	0.10 ± 0.18 ^Aa^
		Yes	0.5	0.0	−0.31 ± 0.04 ^Ba^	−0.41 ± 0.12 ^Ba^	−0.44 ± 0.03 ^Ba^	−0.46 ± 0.03 ^Ba^	−0.51 ± 0.14 ^Ba^	−0.30 ± 0.04 ^Ba^	−0.36 ± 0.00 ^Ba^
		Yes	1	0.0	−0.87 ± 0.12 ^Ba^	−1.06 ± 0.00 ^Cb^	−1.06 ± 0.00 ^Cb^	−1.00 ± 0.03 ^Cb^	−2.50 ± 0.00 ^Cc^	−2.54 ± 0.00 ^Cc^	−3.06 ± 0.00 ^Cd^
				0	3	6	9	12	15	18	21
	10 °C	No	0	0.0	0.14 ± 0.20 ^Ae^	0.46 ± 0.16 ^Ad^	0.50 ± 0.33 ^Ad^	0.77 ± 0.24 ^Ac^	0.96 ± 0.34 ^Ac^	1.52 ± 0.33 ^Ab^	2.00 ± 0.27 ^Aa^*
		No	0.5	0.0	0.00 ± 0.28 ^Ae^	0.14 ± 0.16 ^Ad^	0.18 ± 0.25 ^Bd^	0.39 ± 0.19 ^Bbc^	0.48 ± 0.22 ^Bb^	0.48 ± 0.19 ^Bb^	0.64 ± 0.17 ^Ba^
		No	1	0.0	0.00 ± 0.12 ^Ab^	0.10 ± 0.26 ^Ab^	0.10 ± 0.28 ^Bb^	0.20 ± 0.07 ^Ba^	0.30 ± 0.15 ^Ba^	0.50 ± 0.25 ^Ba^	0.40 ± 0.09 ^Ba^
		Yes	0	0.0	0.10 ± 0.08 ^Ae^	0.23 ± 0.10 ^Acd^	0.44 ± 0.00 ^Ac^	0.60 ± 0.00 ^Abc^	0.90 ± 0.12 ^Ab^	1.12 ± 0.21 ^Aa^	1.50 ± 0.00 ^Aa^
		Yes	0.5	0.0	0.00 ± 0.34 ^Ac^	0.10 ± 0.10 ^Ac^	0.10 ± 0.04 ^Bc^	0.51 ± 0.04 ^Bb^	0.41 ± 0.01 ^Bb^	0.51 ± 0.00 ^Bb^	0.81 ± 0.03 ^Ba^
		Yes	1	0.0	0.00 ± 0.03 ^Aa^	0.00 ± 0.28 ^Aa^	0.10 ± 0.21 ^Ba^	0.20 ± 0.21 ^Ca^	0.21 ± 0.09 ^Ca^	0.30 ± 0.09 ^Ca^	0.40 ± 0.00 ^Ca^
				0	1	2	3	4	5	6	7
	25 °C	No	0	0.0	0.10 ± 0.19 ^Aef^	0.30 ± 0.17 ^Ae^	0.30 ± 0.21 ^Ae^	0.80 ± 0.17 ^Ad^*	1.30 ± 0.37 ^Ac^*	2.00 ± 0.22 ^Ab^*	2.60 ± 0.06 ^Aa^*
		No	0.5	0.0	0.10 ± 0.28 ^Ac^	0.10 ± 0.11 ^Ac^	0.20 ± 0.09 ^Ac^	0.20 ± 0.23 ^Bc^	0.80 ± 0.14 ^Bb^	1.10 ± 0.50 ^BCa^	1.50 ± 0.47 ^BCa^*
		No	1	0.0	0.00 ± 0.17 ^Ab^	0.00 ± 0.39 ^Ab^	0.10 ± 0.38 ^Ab^	0.00 ± 0.06 ^Bb^	0.30 ± 0.14 ^Cb^	0.80 ± 0.26 ^Ca^	0.90 ± 0.29 ^Ca^*
		Yes	0	0.0	0.10 ± 0.01 ^Ae^	0.30 ± 0.00 ^Ad^	0.30 ± 0.17 ^Ad^	0.40 ± 0.00 ^Ad^	1.00 ± 0.00 ^Ac^	1.20 ± 0.02 ^Ab^	1.96 ± 0.00 ^Aa^
		Yes	0.5	0.0	0.00 ± 0.04 ^Ad^	0.30 ± 0.00 ^Bc^	0.10 ± 0.03 ^Bc^	0.21 ± 0.00 ^Bc^	0.62 ± 0.10 ^Bb^	0.90 ± 0.02 ^Ba^	1.00 ± 0.01 ^Ba^
		Yes	1	0.0	0.00 ± 0.10 ^Ab^	0.10 ± 0.12 ^Bb^	0.10 ± 0.07 ^Bb^	0.00 ± 0.15 ^Cb^	0.00 ± 0.03 ^Cb^	0.70 ± 0.34 ^Ca^	0.60 ± 0.34 ^Ca^

Results are mean values of three replications, expressed as log_10_ CFU/g difference compared to the initial time (time 0). Mean values at time 0: 2.28 (with garlic), 2.96 (without garlic). Numbers in the same column with the same uppercase letters are NOT significantly different (*p* > 0.05). Numbers in the same row with the same lowercase letters are NOT significantly different (*p* > 0.05). * Indicates significant difference in effects between the presence and absence of garlic (*p* < 0.05).

**Table 4:** Population changes of *Pseudomonas* spp. in hummus with or without garlic at different chitosan concentrations stored for 28 d at 4 °C, 21 d at 10 °C, and 7 d at 25 °C.

**Temperature**	***Pseudomonas* Counts (log_10_ CFU/g)**										
		**Garlic**	**Chitosan**	**Days**							
				0	4	8	12	16	20	24	28
	4 °C	No	0	0.0	0.18 ± 0.07 ^Ad^	0.35 ± 0.00 ^Ad^	0.46 ± 0.10 ^Acd^	0.62 ± 0.04 ^Ac^	0.70 ± 0.07 ^Ac^	0.81 ± 0.03 ^Aab^	0.95 ± 0.08 ^Aa^
		No	0.5	0.0	0.24 ± 0.12 ^Aa^	0.09 ± 0.09 ^Bab^	0.00 ± 0.21 ^ABab^	−0.28 ± 0.12 ^Bbc^	−0.41 ± 0.06 ^Bc^	−0.52 ± 0.21 ^Bc^	−1.00 ± 0.21 ^Bd^
		No	1	0.0	−0.09 ± 0.21 ^Aa^	−0.24 ± 0.00 ^Cb^	−0.41 ± 0.18 ^Bb^	−0.92 ± 0.00 ^Cc^*	−1.04 ± 0.00 ^Cc^*	−1.24 ± 0.00 ^Cd^*	−2.00 ± 0.00 ^Ce^*
		Yes	0	0.0	0.10 ± 0.10 ^Acd^	0.21 ± 0.04 ^Ac^	0.32 ± 0.04 ^Ac^	0.52 ± 0.11 ^Ab^	0.70 ± 0.09 ^Aab^	0.92 ± 0.08 ^Aa^	1.00 ± 0.32 ^Aa^
		Yes	0.5	0.0	0.12 ± 0.13 ^Aa^	0.10 ± 0.21 ^Aa^	0.20 ± 0.17 ^Aa^	−0.20 ± 0.12 ^Bb^	−0.61 ± 0.13 ^Bbc^	−0.88 ± 0.14 ^Bc^	−1.55 ± 0.15 ^Bd^
		Yes	1	0.0	0.0 ± 0.09 ^Aa^	−0.10 ± 0.14 ^Aa^	−0.32 ± 0.32 ^Bb^	−1.24 ± 0.03 ^Cc^	−1.55 ± 0.04 ^Cc^	−2.24 ± 0.15 ^Cd^	−2.24 ± 0.30 ^Cd^
				0	3	6	9	12	15	18	21
	10 °C	No	0	0.0	0.20 ± 0.21 ^Ad^	0.35 ± 0.00 ^Ad^	0.70 ± 0.07 ^Ac^	0.85 ± 0.14 ^Ac^	1.05 ± 0.00 ^Ab^*	1.68 ± 0.04 ^Aa^*	2.20 ± 0.21 ^Aa^*
		No	0.5	0.0	−0.11 ± 0.06 ^Bbc^	0.00 ± 0.21 ^Bb^	0.07 ± 0.06 ^Bb^	0.08 ± 0.04 ^Bb^	0.26 ± 0.10 ^Ba^	0.36 ± 0.05 ^Ba^	0.44 ± 0.02 ^Ba^
		No	1	0.0	−0.14 ± 0.20 ^Ba^	0.00 ± 0.21 ^Ba^	−0.09 ± 0.24 ^Ca^	−0.07 ± 0.06 ^Ca^	−0.02 ± 0.13 ^Ca^	−0.03 ± 0.10 ^Ca^	0.01 ± 0.15 ^BCa^
		Yes	0	0.0	0.10 ± 0.03 ^Ad^	0.29 ± 0.02 ^Ad^	0.55 ± 0.13 ^Ac^	0.75 ± 0.12 ^Ab^	0.95 ± 0.01 ^Aa^	1.02 ± 0.27 ^Aa^	1.95 ± 0.19 ^Aa^
		Yes	0.5	0.0	−0.15 ± 0.18 ^Bc^	0.00 ± 0.06 ^Bc^	0.05 ± 0.06 ^Bbc^	0.10 ± 0.21 ^Bb^	0.31 ± 0.09 ^Bab^	0.50 ± 0.01 ^Ba^	0.52 ± 0.05 ^Ba^
		Yes	1	0.0	−0.25 ± 0.01 ^Ba^	−0.10 ± 0.01 ^Ba^	−0.10 ± 0.07 ^Ba^	−0.20 ± 0.02 ^Ca^	−0.10 ± 0.28 ^Ca^	−0.20 ± 0.40 ^Ca^	−0.30 ± 0.01 ^Ca^
				0	1	2	3	4	5	6	7
	25 °C	No	0	0.0	0.40 ± 0.12 ^Af^	1.40 ± 0.00 ^Ae^*	1.90 ± 0.21 ^Ad^*	2.40 ± 0.12 ^Ac^*	2.90 ± 0.22 ^Ab^*	3.00 ± 0.00 ^Ab^*	3.40 ± 0.12 ^Aa^*
		No	0.5	0.0	0.20 ± 0.00 ^ABc^	0.60 ± 0.0 6 ^Bbc^	0.80 ± 0.00 ^Bab^	0.80 ± 0.00 ^Bab^	1.10 ± 0.25 ^Bab^	1.20 ± 0.14 ^Ba^	1.30 ± 0.43 ^Ba^
		No	1	0.0	−0.10 ± 0.21 ^Bd^	0.40 ^Cc^ ± 0.00	0.50 ± 0.00 ^Bbc^	0.70 ± 0.04 ^Bab^	0.80 ± 0.03 ^Bc^	0.80 ± 0.08 ^Cc^	0.80 ± 0.05 ^Bc^
		Yes	0	0.0	0.30 ± 0.30 ^Af^	0.60 ± 0.04 ^Ae^	1.00 ± 0.02 ^Ad^	1.56 ± 0.06 ^Ac^	2.00 ± 0.03 ^Abc^	2.50 ± 0.43 ^Aa^	2.90 ± 0.04 ^Aa^
		Yes	0.5	0.0	0.10 ± 0.04 ^Ac^	0.20 ± 0.26 ^Bc^	0.30 ± 0.20 ^Bc^	0.50 ± 0.06 ^Bb^	0.95 ± 0.02 ^Ba^	1.00 ± 0.02 ^Ba^	1.10 ± 0.04 ^Ba^
		Yes	1	0.0	−0.10 ± 0.01 ^Ac^	0.20 ± 0.30 ^Bc^	0.20 ± 0.05 ^Bbc^	0.30 ± 0.22 ^Bb^	0.50 ± 0.02 ^Ba^	0.49 ± 0.03 ^Ca^	0.68 ± 0.04 ^Ca^

Results are mean values of three replications, expressed as log_10_ CFU/g differences compared to the initial time (time 0). Mean values at time 0: 2.28 (with garlic), 2.96 (without garlic). Numbers in the same column with the same uppercase letters are NOT significantly different (*p* > 0.05). Numbers in the same row with the same lowercase letters are NOT significantly different (*p* > 0.05). * Indicates significant difference in effects between the presence and absence of garlic (*p* < 0.05).

**Table 5:** Change in pH at 4 °C, 10 °C, and 25 °C.

Temperature	Garlic	Chitosan	Days							
			0	4	8	12	16	20	24	28
4	No	0.00	4.49 ± 0.01 ^C^	4.41 ± 0.20 ^E^	4.39 ± 0.01 ^DE^	4.40 ± 0.09 ^DE^	4.50 ± 0.04 ^ABCDE^	4.42 ± 0.01 ^AB^	4.25 ± 0.03 ^AB^	4.00 ± 0.01 ^BCDE^
4	No	0.50	4.84 ± 0.02 ^B^	4.46 ± 0.06 ^DE^	4.43 ± 0.01 ^DE^	4.75 ± 0.06 ^ABCD^	4.60 ± 0.08 ^ABC^	4.29 ± 0.02 ^CD^	4.30 ± 0.03 ^AB^	4.10 ± 0.25 ^ABC^
4	No	1.00	4.91 ± 0.02 ^A^	4.48 ± 0.18 ^CDE^	4.53 ± 0.02 ^D^	4.91 ± 0.03 ^ABC^	4.81 ± 0.05 ^A^	4.46 ± 0.05 ^AB^	4.51 ± 0.07 ^A^	4.20 ± 0.05 ^AB^
4	Yes	0.00	4.56 ± 0.04 ^C^	4.45 ± 0.06 ^DE^	4.33 ± 0.05 ^E^	4.42 ± 0.06 ^DE^	4.70 ± 0.04 ^AB^	4.47 ± 0.03 ^AB^	4.53 ± 0.05 ^A^	4.40 ± 0.01 ^A^
4	Yes	0.50	4.75 ± 0.04 ^B^	4.55 ± 0.05 ^CDE^	4.40 ± 0.12 ^DE^	4.67 ± 0.02 ^ABCDE^	4.60 ± 0.02 ^ABC^	4.37 ± 0.07 ^BC^	4.15 ± 0.05 ^BCD^	4.10 ± 0.03 ^BCD^
4	Yes	1.00	4.84 ± 0.04 ^B^	4.40 ± 0.01 ^E^	4.50 ± 0.09 ^D^	4.95 ± 0.03 ^AB^	4.40 ± 0.03 ^ABCDEF^	4.51 ± 0.01 ^A^	4.30 ± 0.30 ^AB^	4.09 ± 0.03 ^BCD^
			0	3	6	9	12	15	18	21
10	No	0.00	4.49 ± 0.01 ^C^	4.40 ± 0.03 ^E^	4.41 ± 0.07 ^DE^	4.40 ± 0.14 ^DE^	4.55 ± 0.35 ^ABCD^	4.10 ± 0.06 ^EF^	4.00 ± 0.02 ^BCDE^	3.80 ± 0.07 ^E^
10	No	0.50	4.84 ± 0.02 ^B^	4.61 ± 0.05 ^BCDE^	4.77 ± 0.06 ^C^	4.70 ± 0.06 ^ABCDE^	4.30 ± 0.01 ^BCDEF^	4.00 ± 0.03 ^FG^	3.90 ± 0.09 ^CDEF^	3.90 ± 0.02 ^CDE^
10	No	1.00	4.91 ± 0.02 ^A^	4.88 ± 0.03 ^A^	5.00 ± 0.03 ^A^	5.00 ± 0.03 ^A^	4.41 ± 0.09 ^ABCDEF^	3.90 ± 0.08 ^GH^	3.80 ± 0.04 ^FGH^	3.50 ± 0.02 ^F^
10	Yes	0.00	4.56 ± 0.04 ^C^	4.40 ± 0.03 ^E^	4.40 ± 0.04 ^DE^	4.50 ± 0.05 ^DE^	4.15 ± 0.02 ^DEF^	4.00 ± 0.06 ^FG^	4.10 ± 0.04 ^BCDE^	4.00 ± 0.01 ^BCDE^
10	Yes	0.50	4.75 ± 0.04 ^B^	4.67 ± 0.05 ^ABCD^	4.70 ± 0.07 ^C^	4.95 ± 0.17 ^AB^	4.30 ± 0.05 ^BCDEF^	4.20 ± 0.09 ^DE^	4.20 ± 0.02 ^ABC^	4.10 ± 0.01 ^BCD^
10	Yes	1.00	4.84 ± 0.04 ^B^	4.90 ± 0.08 ^A^	4.98 ± 0.05 ^A^	4.63 ± 0.06 ^BCDE^	4.25 ± 0.04 ^CDEF^	4.00 ± 0.03 ^FG^	4.00 ± 0.00 ^BCDE^	4.20 ± 0.01 ^AB^
			0	1	2	3	4	5	6	7
25	No	0.00	4.49 ± 0.01 ^C^	4.48 ± 0.05 ^CDE^	4.94 ± 0.02 ^AB^	4.36 ± 0.14 ^E^	4.36 ± 0.16 ^BCDEF^	4.00 ± 0.02 ^FG^	3.48 ± 0.08 ^HIJ^	3.48 ± 0.08 ^F^
25	No	0.50	4.84 ± 0.02 ^B^	4.70 ± 0.05 ^ABC^	4.29 ± 0.06 ^E^	4.57 ± 0.32 ^CDE^	4.01 ± 0.01 ^F^	3.90 ± 0.03 ^GH^	3.35 ± 0.15 ^IJ^	3.35 ± 0.15 ^F^
25	No	1.00	4.91 ± 0.02 ^A^	4.81 ± 0.03 ^AB^	4.08 ± 0.03 ^F^	4.63 ± 0.09 ^BCDE^	4.23 ± 0.28 ^CDEF^	3.80 ± 0.03 ^H^	3.25 ± 0.15 ^JK^	3.25 ± 0.15 ^F^
25	Yes	0.00	4.56 ± 0.04 ^C^	4.60 ± 0.06 ^BCDE^	4.33 ± 0.04 ^E^	4.70 ± 0.01 ^ABCDE^	4.05 ± 0.05 ^F^	4.10 ± 0.01 ^EF^	2.95 ± 0.05 ^K^	2.95 ± 0.05 ^G^
25	Yes	0.50	4.75 ± 0.04 ^B^	4.70 ± 0.03 ^ABC^	4.78 ± 0.04 ^BC^	4.68 ± 0.19 ^ABCDE^	4.09 ± 0.12 ^EF^	4.00 ± 0.01 ^FG^	3.85 ± 0.16 ^DEF^	3.85 ± 0.15 ^DE^
25	Yes	1.00	4.84 ± 0.04 ^B^	4.80 ± 0.06 ^AB^	4.03 ± 0.03 ^F^	4.67 ± 0.03 ^ABCDE^	4.31 ± 0.21 ^BCDEF^	3.90 ± 0.04 ^GH^	3.64 ± 0.17 ^GHI^	4.14 ± 0.39 ^ABC^

Numbers in the same column with the same uppercase letters are NOT significantly different (*p* > 0.05).

**Table 6:** The color was tested among the different treated hummus samples with garlic and chitosan.

Particulars	CNL	No Garlic+ 0.5% Chitosan	No Garlic+ 1.0% Chitosan	+ 1% Garlic+ No Chitosan	1% Garlic+ 0.5% Chitosan	1% Garlic+ 1.0% Chitosan	*p*-Value
l*	79.65 ± 0.06	79.51 ± 0.18	79.21 ± 0.57	79.50 ± 0.11	79.56 ± 0.36	79.42 ± 0.25	0.625
a*	1.12 ^a^ ± 0.04	1.08 ^a^ ± 0.01	0.88 ^b^ ± 0.09	1.15 ^a^ ± 0.02	1.13 ^a^ ± 0.01	1.13 ^a^ ± 0.02	<0.001
b*	19.64 ^a^ ± 0.03	19.44 ^ab^ ± 0.02	19.12 ^c^ ± 0.16	19.45 ^ab^ ± 0.08	19.36 ^b^ ± 0.47	19.37 ^b^ ± 0.04	<0.001

Numbers in the same column with the same uppercase letters are NOT significantly different (*p* < 0.001).

**Table 7:** TBAR (Conc. (MDA Eq/kg of sample)) was tested among the different treated hummus samples with garlic and chitosan.

Particulars	CNL	No Garlic+ 0.5% Chitosan	No Garlic+ 1.0% Chitosan	+1% Garlic+ No Chitosan	1% Garlic+ 0.5% Chitosan	1% Garlic+ 1.0% Chitosan	*p*-Value
TBAR (Conc (MDA Eq/kg of sample))	5.42 ^b^ ± 0.05	6.85 ^a^ ± 0.21	5.72 ^b^ ± 0.05	4.00 ^d^ ± 0.03	4.37 ^cd^ ± 0.24	4.64 ^c^ ± 0.08	*p* < 0.001

Numbers in the same column with the same uppercase letters are NOT significantly different (*p* < 0.001).

**Table 8:** Textural profile of hummus with or without garlic at different chitosan concentrations.

Sample	Hardness	Adhesiveness (mJ)	Stringiness (mm)
500 g of Hummus Samples + No Garlic + No chitosan	96.9 ± 14.8 ^c^	4.2 ± 1.1 ^a^	8.4 ± 2.2 ^c^
500 g of Hummus Samples + No Garlic + 0.5% chitosan	98.9 ± 12.7 ^b^	4.4 ± 1.1 ^a^	8.7 ± 1.9 ^b^
500 g of Hummus Samples + No Garlic + 1.0% chitosan	100.1 ± 13.7 ^b^	4.6 ± 0.9 ^a^	9.0 ± 1.6 ^a^
500 g of Hummus Samples + 1% Garlic + No chitosan	99.8 ± 10.9 ^b^	4.5 ± 0.9 ^a^	9.3 ± 1.3 ^a^
500 g of Hummus Samples + 1% Garlic + 0.5% chitosan	104.3 ± 16.2 ^ab^	4.5 ± 1.0 ^a^	8.5 ± 1.6 ^c^
500 g of Hummus Samples + 1% Garlic + 1.0% chitosan	108.0 ± 11.8 ^a^	4.1 ± 0.9 ^a^	8.7 ± 1.5 ^b^

Numbers in the same column with the same lowercase letters are NOT significantly different (*p* > 0.05).

## Data Availability

The original contributions presented in the study are included in the article, further inquiries can be directed to the corresponding author.
